# Dog Poisonings Associated with a *Microcystis aeruginosa* Bloom in the Netherlands

**DOI:** 10.3390/toxins5030556

**Published:** 2013-03-14

**Authors:** Miquel Lürling, Elisabeth J. Faassen

**Affiliations:** 1 Aquatic Ecology & Water Quality Management Group, Wageningen University, P.O. Box 47, Wageningen 6700 DD, The Netherlands; E-Mail: els.faassen@wur.nl; 2 NIOO-KNAW, Droevendaalsesteeg 10, Wageningen 6708 PB, The Netherlands

**Keywords:** bathing water, cyanobacterial scum, cyanotoxins, microcystin, LC-MS/MS, swimming ban

## Abstract

In early autumn 2011, three dogs died after they had been exposed to a *Microcystis aeruginosa* bloom on Lake Amstelmeer, The Netherlands. The cyanobacterial scum from the lake contained up to 5.27 × 10^3^μg g^−1^ dry-weight microcystin, the vomit of one of the dogs contained on average 94 µg microcystin g^−1^ dry-weight. In both cases, microcystin-LR was the most abundant variant. This is the first report of dog deaths associated with a *Microcystis* bloom and microcystin poisoning in The Netherlands.

## 1. Introduction

Eutrophication of surface waters has many undesirable effects and has become the major water quality issue in many freshwater and coastal systems world wide [1]. Cyanobacterial blooms are one symptom of eutrophication. These blooms present a serious threat to the environment and the health of wildlife, cattle, pets and humans because of the ability of cyanobacteria to produce potent toxins [2,3].

Numerous animal poisonings associated with cyanobacterial blooms have been documented and among these are several cases of dog deaths [4]. Dogs seem to be attracted by the odors produced by cyanobacteria and might swallow substantial amounts of floating mats accumulated on leeside shorelines [5]. Indeed, many dog fatalities have been attributed to consumption of benthic cyanobacterial mat material, mostly dominated by *Oscillatoria* and *Phormidium* spp. [6,7,8,9]. In these cases, anatoxin-a or homoanatoxin-a poisoning was identified as the most likely causal factor. In line with those findings, the death of three dogs that ingested *Phormidium* mat material that had been washed ashore at Lake IJmeer (The Netherlands) in spring 2011, seemed also to be caused by (homo)anatoxin-a poisoning [10]. However, blooms of pelagic cyanobacteria are also linked to dog poisonings: dogs deaths associated with *Nodularia* blooms have been described from the Australian lake Alexandrina [11] and the Baltic Sea [12,13]. In addition, three dogs died of possible *Microcystis* poisoning in Baptist lake, Northern Alberta [14] and a representative of the same genus was held responsible for the death of six dogs in Qu’Apelle Lake, Saskatchewan [15].

Some months after the dog deaths in spring 2011 in Lake IJmeer [10], another incident led to the death of three dogs that had been swimming in the Dutch Lake Amstelmeer. At the time of the dog fatalities, Lake Amstelmeer experienced a massive bloom of the cyanobacterium *Microcystis aeruginosa.* This species is one of the most frequently encountered bloom-forming cyanobacteria in freshwater bodies all around the world [16,17]. The most notorious toxins produced by toxigenic *M. aeruginosa* strains are microcystins (MC), which are microbial non-ribosomal processed cyclic heptapeptides [18]. We analyzed cyanobacterial samples from Lake Amstelmeer, and the vomit of one of the deceased dogs, a Labrador Retriever, for microcystins and report on microcystin poisoning as a plausible cause of the dogs’ death.

## 2. Results and Discussion

On 29 September 2011, a Labrador Retriever of about 30 kg and a 16 weeks old Jack Russell pup were brought to the regional veterinary hospital (Veterinair Centrum Holland Noord, Slootdorp, The Netherlands) after they had been walked on the shore of Lake Amstelmeer (The Netherlands). The Labrador Retriever had consumed cyanobacterial scum material that had been washed on the shore. The dog vomited severely, became lethargic, showed difficulties in breathing and died after four to five hours. The vomit of this dog was collected. The Jack Russell pup had not been eating from the scum material on the shore, but had been swimming in the scum. Also this dog vomited and died after 12 to 16 h. A few days later, a second Labrador Retriever was brought to the veterinary hospital. Also this dog had been swimming in Lake Amstelmeer, after which it lost appetite, became lethargic, showed difficulties in moving, signs of abdominal pain, indications of gastro-intestinal bleedings and shallow breathing before it died. The haematocrit level of this dog (26.5%) was strongly reduced compared to baseline levels in Labrador Retrievers, which is approximately 44% [19].

**Figure 1:**
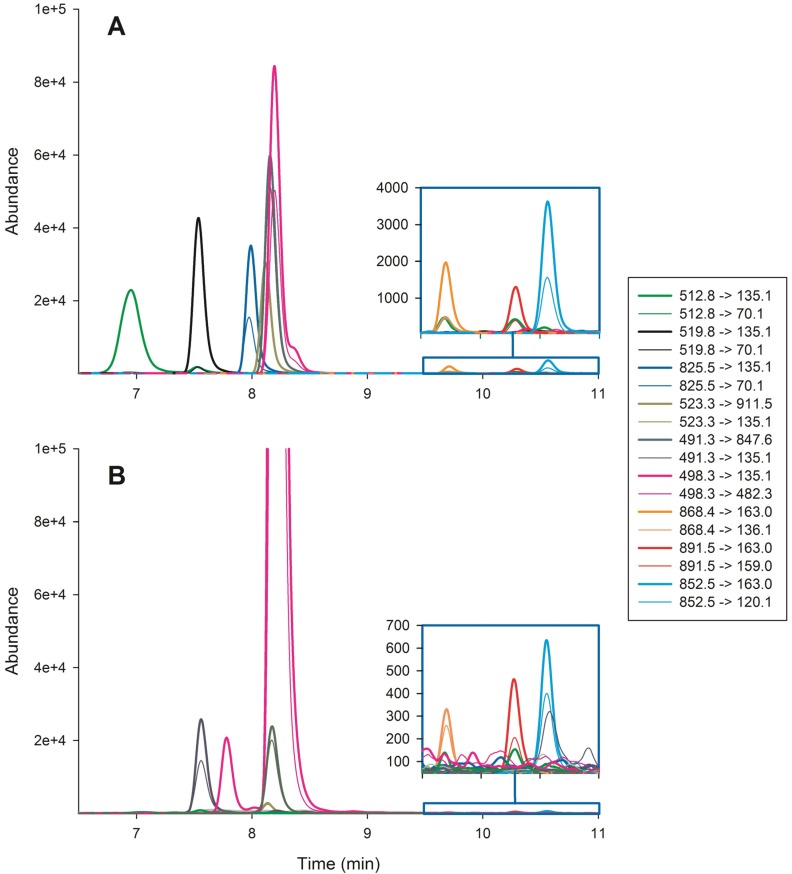
Chromatogram of (**A**) a calibration standard containing eight microcystins and nodularin and (**B**) an undiluted sample of dog vomit. Transitions for the same compounds are shown in the same color, transition for the quantifier ion are represented by a bold line, transitions for the qualifier ions are represented by a normal line.

The water and scum samples from Lake Amstelmeer contained the cyanobacterium *Microcystis aeruginosa*, which was identified microscopically and by 16S rRNA analysis (Supplementary Information 1). The vomit of the first Labrador Retriever also contained *Microcystis* like cells and small multi-celled *Microcystis* aggregates, as determined by light microscopy. Because the cyanobacterial bloom consisted of *Microcystis*, the cyanobacterial samples and the vomit were subjected to microcystin (MC) analysis. Chromatograms of a calibration standard and an undiluted vomit sample are shown in Figure 1. 

**Figure 2:**
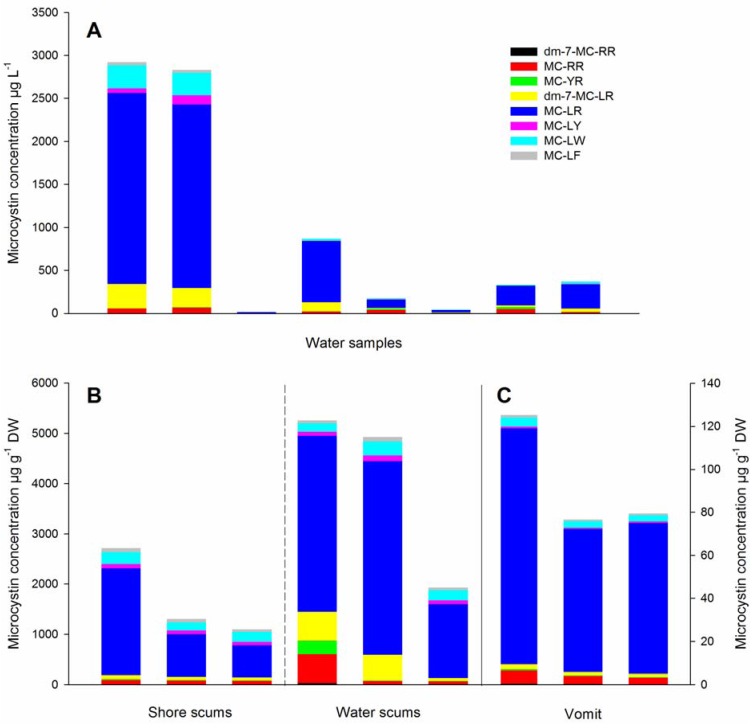
Concentrations of eight microcystin variants in (**A**) water samples collected at eight different sites in Lake Amstelmeer, The Netherlands; (**B**) scum material collected at the shore and from the surface of the lake and (**C**) vomit of a Labrador retriever that died after ingesting scum material. Each part of each column represents the average of three analytical replicates, full data are presented in Supplementary Information 2.

MC data for each sample and variant are listed in Supplementary Information 2 and are summarized in Figure 2. All samples contained MCs, the total MC concentration in water samples from Lake Amstelmeer was different in different locations, it ranged from 17 to 2.92 × 10^3^ µg L^−1^ (Figure 2A). The scum material that was collected at the shore contained on average less MCs (1.71 × 10^3^ µg g^−1^ dry-weight) than the scums collected from the water surface (4.04 × 10^3^ µg g^−1^ dry-weight, Figure 2B). The vomit of the dog contained roughly 5% (94 µg g^−1^ dry-weight) of the MC content of the shore scums (Figure 2C). None of the samples contained detectable amounts of NOD.

The samples contained all eight analyzed MC variants and in all cases MC-LR was most abundant, on average it made up 73% of the total MC content (Figure 2). MC-RR was on average present in 7.5%, MC-LW in 6.6% and dm-7-MC-LR in 6.0% of the total identified MCs (Figure 2). 

The variant profiles of the shore scum and the dog’s vomit were similar, with MC-LR being slightly more abundant in the vomit (Figure 2). As the dog had ingested cyanobacterial material from the shore, this similarity of the variant profiles was expected. The variation in variant composition of the water samples and the water scums was larger than for the shore scums and the dog vomit (Figure 2). These larger variations seem not to be caused by analytical differences, since all samples consist of the same type of matrix, so matrix effect are expected to be the same between these samples. Instead, it is more likely that these differences are caused by spatial heterogeneity of the cyanobacterial bloom. Within one lake, cyanobacterial abundance and species composition change in time and space and a bloom can consist of different genotypes of the same species, which may produce different MC variants but cannot be distinguished by microscopy [20,21]. Moreover single genotypes can alter the relative abundances of the MC variants they produce as a response to locally changing availability of nutrients [22].

The more hydrophobic variants MC-LW and MC-LF were present in lower concentrations than MC-LR, but recent *in vitro* studies indicate that these two variants might be more toxic [23,24,25]. So although the combined average abundance of MC-LW and MC-LF was 12% of that of MC-LR, their assumed higher toxicity (the most conservative estimate from [25] is 6.8 times that of MC-LR) makes their contribution to the total toxicity at least of the same order of magnitude as that of MC-LR, but possibly higher.

The results of this study point towards MC poisoning as the cause of the dogs’ deaths. Water samples, scum samples and vomit of one of the ceased dogs all contained *Microcystis* and MCs. Furthermore, the dogs symptoms, such as vomiting, becoming lethargic, signs of abdominal pain and neurological disorders, resemble those that have been described for patients from a dialysis center in Brazil who had been exposed to MC contaminated dialysis water [26].

The *M. aeruginosa* from Lake Amstelmeer contained very high concentrations of MCs. The highest value found for one of the surface scums was 5.27 × 10^3^µg MC g^−1^ dry-weight, placing it among the highest MC concentrations measured in scums worldwide (Table 1). The water samples showed a substantial variation in MC concentrations (17–2.92 × 10^3^µg L^−1^), which might reflect considerable spatial heterogeneity in *Microcystis* abundance [27,28]. Moreover, the bloom occurred end of September–beginning of October, illustrating that high MC concentrations caused by *Microcystis* blooms in temperate regions of Europe are not restricted to the summer months July and August [29,30].

**Table 1:** Microcystin (MC) concentrations of cyanobacterial bloom material from different sites and studies.

Lake (country)	MC Concentration (µg g^−1^ dry-weight)	reference
Lake Baringo (Kenya)	19800	[31]
Bautzen Reservoir (Germany)	14700	[32]
Beaver Dam Lake 2 (USA)	12800	[33]
Lake Winnebago 1 (USA)	10240	[33]
Lalla Takerkoust (Morocco)	8800	[34]
Fish pond S2 Wuhan (China)	7280	[35]
River Guadiana (Portugal)	7100	[36]
Not specified (Germany)	5595	[37]
Lake Amstelmeer (Netherlands)	5265	This study
Lake Grand-Lieu (France)	5060	[38]
Lake Oubeira (Algeria)	4590	[39]
Laguna de Bay (Philippines)	4049	[40]

In conclusion, three dogs likely died as a result of exposure to MCs from a *M. aeruginosa* bloom in Lake Amstelmeer. This is the first report of MC poisoning of dogs in The Netherlands.

## 3. Experimental Section

Lake Amstelmeer is located in the northwest of The Netherlands (52°52'N 4°54'E). The 650 ha lake was created after damming the former estuary in 1930 and subsequently deepened by sand excavation giving it a maximum depth of 18 m. The water is light brackish (580–1600 mg chloride L^−1^) and suffers regularly from cyanobacterial blooms [41]. Just after the dog fatalities at Lake Amstelmeer end September–beginning October 2011, scum material was collected at various locations on the water surface and at the shoreline and water samples were taken at eight different sites in the lake. Vomit (403 g wet weight) was collected from a Labrador Retriever of about 30 kg who died September 29th 2011 after ingesting cyanobacterial scum material.

The cyanobacterial samples and the vomit of the Labrador Retriever were inspected microscopically using a Nikon light microscope at 750× magnification. Freeze dried cyanobacterial material was send to Baseclear BV (Leiden, The Netherlands) for 16S rRNA analysis. About 700 base pairs of the 16S rRNA gene were amplified and sequenced on both strands. Sequence was analyzed using the Integrated Database Network System (IDNS) SmartGene 16S rRNA eubacteria database (Baseclear BV). 

Vomit and scum material were prepared for microcystin (MC) analysis by freeze-drying. From each sample/location, aliquots of 5 mg freeze-dried material were transferred in triplicate to 2 mL Eppendorf vials (biological replicates). MCs were extracted three times at 60 °C in 0.5 mL 75% methanol-25% Millipore water (Billerica, MA, USA) (*v*/*v*). Extracts were dried in a Speedvac (Thermo Scientific Savant SPD121P, Asheville, NC, USA) and reconstituted in 600 μL methanol. The reconstituted samples were transferred to 2 mL Eppendorf vials with a cellulose-acetate filter (0.2 μm, Grace Davison Discovery Science, Columbia, SC, USA) and centrifuged for 5 min at 16,000 × *g* (VWR Galaxy 16DH, Boxmeer, The Netherlands). Filtrates were transferred to amber glass vials before analysis.

Water samples were glass-fiber filtered (Whatman GF/C, Buckinghamshire, UK) and stored overnight at −20 °C. The frozen filters were extracted and processed as described in [30]. 

Calibration standards for all analyzed compounds were obtained from DHI LAB products (Hørsholm, Denmark, Table 2). 

**Table 2:** Calibration standard details for microcystins (MC) and nodularin (NOD) and composition of the amino acids on position 2 and 4.

Compound	Position 2	Position 4
dm-7-MC-RR ^1^	Arginine	Arginine
MC-RR	Arginine	Arginine
NOD	n.a.	n.a.
MC-YR	Tyrosine	Arginine
dm-7-MC-LR ^1^	Leucine	Arginine
MC-LR	Leucine	Arginine
MC-LY	Leucine	Tyrosine
MC-LW	Leucine	Tryptophan
MC-LF	Leucine	Phenylalanine

^1^ dm = desmethylated.

LC-MS/MS analysis was performed on an Agilent 1200 LC and an Agilent 6410A QQQ (Waldbronn, Germany). The compounds were separated on an Agilent Zorbax Eclipse XDB-C18 (Santa Clara, CA, USA) 4.6 × 150 mm, 5 μm column by Millipore water with 0.1% formic acid (*v*/*v*, eluent A) and acetonitrile with 0.1% formic acid (*v*/*v*, eluent B). Elution program was 0–2 min 30% B, 6–12 min 90% B, with a linear increase of B between 2 and 6 min and a 5 min post run at 30% B. Injection volume was 10 µL, flow 0.5 mL min^−1^, column temperature was 40 °C. The LC-MS/MS was operated in positive mode with an ESI source, nitrogen was used as drying and collision gas. For each compound, two transitions were monitored in MRM mode. The first quadrupole was operated in unit mode, the second quadrupole was operated in widest mode. Dwell time was 50 ms. Eight MC variants and nodularin (NOD) were analysed, MS/MS settings are shown in Table 3. 

**Table 3:** MS/MS settings for microcystin (MC) and nodularin (NOD) analysis.

Compound	Retention time (min)	Precursor ion (*m/z*)	Fragmentor (V)	Quantifier ion (*m/z*)	CE ^1^ quantifier (V)	Qualifier ion (*m/z*)	CE ^1^ qualifier (V)	Ratio ^2^ (%)
dm-7-MC-RR	6.93	512.8	135	135.1	26	70.1	85	1.2
MC-RR	7.62	519.8	151	135.1	30	70.1	75	2.7
NOD	8.03	825.5	220	135.1	70	70.1	95	44.2
MC-YR	8.16	523.3	102	911.5	5	135.1	6	103.6
dm-7-MC-LR	8.21	491.3	88	847.6	5	135.1	6	84.0
MC-LR	8.24	498.3	88	135.1	6	482.3	6	56.7
MC-LY	9.67	868.4	170	163.0	35	136.1	75	29.0
MC-LW	10.22	891.5	146	163.0	31	159.0	75	26.9
MC-LF	10.47	852.5	140	163.0	31	120.1	79	39.1

^1^ collision energy, ^2^ ratio between abundance of the qualifier and quantifier ion.

Recovery of sample workup and analysis was determined by spiking a cyanobacterial matrix in triplicate and was between 54% for MC-LW and 105% for NOD (Table 4). Each sample was injected in triplicate (technical replicates). Samples were quantified against a calibration curve in methanol and subsequently corrected for recoveries. When necessary, samples were diluted in methanol until they fell within the calibration range. Limit of detection (LOD) in calibration standards was defined as the lowest injected concentration with a signal-to-noise (*S*/*N*) ratio of both product ions of at least 3:1. Furthermore, the ratio of the qualifier ions to the quantifier ion should be within a 20% relative range of the expected value (Table 3). Limit of quantification (LOQ) was defined as the lowest injected concentration with a *S*/*N* ratio of the quantifier ion of at least 10:1. Furthermore, the ratio of the qualifier ion to the quantifier should again be within the accepted range, and the *S*/*N* ratio of the qualifier ion should at least be 3:1. For some variants, the LOD then equaled LOQ (Table 4) because the conditions for the ratio of the qualifier ions to the quantifier ion or for the *S*/*N* of the qualifier ions were sometimes only met at a concentration where the S/N ratio of the quantifier ion is at least 10:1. Detection limits and calibration curve range are shown in Table 4. The calibration curves of dm-7-MC-RR and MC-RR were slightly quadratic.

**Table 4:** Quantification details of microcystins (MC) and nodularin (NOD) LC-MS/MS analysis.

Compound	LOD ^1^ (fmole inj^−1^)	LOQ ^2^ (fmole inj^−1^)	Calibration curve range (μg L^−1^)	Curve shape	Recovery (%)
dm-7-MC-RR	132	132	14–338	Quadratic	100
MC-RR	92	92	10–949	Quadratic	96
NOD	<17	17	1–368	Linear	105
MC-YR	<10	10	1–518	Linear	75
dm-7-MC-LR	<12	12	1–589	Linear	78
MC-LR	<19	19	2–921	Linear	79
MC-LY	165	165	16–824	Linear	73
MC-LW	77	154	16–791	Linear	54
MC-LF	37	37	4–900	Linear	64

^1^ Limit of detection, ^2^ Limit of quantification.

Repeatability was determined by ten subsequent injections of the same calibration standards, results are shown in Table 5.

**Table 5:** Repeatability of microcystins (MC) and nodularin (NOD) LC-MS/MS analysis, expressed in relative standard deviations (%), *n* = 10.

Compound	Retention time	Peak area ^1^	Ratio ^2^
dm-7-MC-RR	0.6	5.4	5.8
MC-RR	0.1	6.6	6.1
NOD	0.1	3.7	1.5
MC-YR	0.0	1.3	0.9
dm-7-MC-LR	0.1	1.5	1.4
MC-LR	0.0	1.4	2.8
MC-LY	0.1	1.6	7.2
MC-LW	0.1	3.4	11.2
MC-LF	0.0	4.9	7.4

^1^ area of the quantifier ion; ^2^ ratio between abundance of the qualifier and quantifier ion.

For dm-7-MC-RR and MC-RR, the ratio between the quantifier ion and the qualifier ion as listed in Table 3 was not constant, in calibration standards it increased with increasing concentration, and in the presence of a matrix, it increased up to 40%. As this also occurred when cyanobacterial samples were spiked with these compounds, ratios between those listed in Table 3 and 40% were accepted in samples. Finally, the ratio between the quantifier ion and the qualifier ion of MC-LY was increased in concentrated samples (Figure 1B). When samples were diluted, the ratio returned to the expected value. If necessary, the presence of MC-LY was therefore confirmed in diluted samples.

## 4. Conclusions

A *Microcystis aeruginosa* bloom in Lake Amstelmeer (The Netherlands) contained very high microcystin (MC) concentrations up to 5.27 × 10^3^µg g^−1^ dry-weight in material accumulated on the water surface and shores. 

As vomit of one of the deceased dogs contained MCs and all casualties had ingested accumulated *Microcystis* material or had been exposed to it, the three dogs likely died as a result of exposure to MCs from the *M. aeruginosa* bloom in Lake Amstelmeer. 

This is the first report of MC poisoning of dogs in The Netherlands.

## Supplementary Files

Supplementary File 1 SI 1 Results genetic identification (PDF, 2424 KB)

Supplementary File 2 SI 2 Tables MC concentrations (PDF, 81 KB)
