# Supplementary material for: Dog Poisonings Associated with a Microcystis aeruginosa Bloom in the Netherlands
Source: Toxins (Basel). 2013 Mar 14;5(3):556–67. doi: 10.3390/toxins5030556 (PMC3705278; doi:10.3390/toxins5030556)
Supplement: Supplementary File 1 — SI 1 Results genetic identification (PDF, 2424 KB) [file toxins-05-00556-s001.pdf]

# Sequence-based Strain Identification Report

| **Sample name** | AMS 5-10-11 | **Bact code** | 101263 | **Project code** | 52436 |
|---|---|---|---|---|---|
| **Sample creation** | 18.07.2012 | **Sample update** | 18.07.2012 | | |

# Result

| **Final identification:** | **Microcystis aeruginosa, Microcystis ichthyoblabe** |
|---|---|
| Closest reference found: | Microcystis aeruginosa AF139305 702 (100.00%) |
| Remark: | Microcystis ichthyoblabe AB012338 702 (100.00%) |

## Similarity search summary

Scanned databases (Db):
D1 - IDNS 16S rDNA Eubacteria

| Rank | Db | AC - Accession | OS - Organism | Seq. Length | Identities | Mismatches | Match Length | Score |
|---|---|---|---|---|---|---|---|---|
| 1 | D1 | AF139305 | Microcystis aeruginosa | 1421 | 702 (100.00%) | 0 | 702 | 742.0 |
| 2 | D1 | AF139320 | Microcystis aeruginosa | 1421 | 702 (100.00%) | 0 | 702 | 742.0 |
| 3 | D1 | AF139323 | Microcystis aeruginosa | 1421 | 702 (100.00%) | 0 | 702 | 742.0 |
| 4 | D1 | AF139292 | Microcystis aeruginosa | 1421 | 702 (100.00%) | 0 | 702 | 742.0 |
| 5 | D1 | DQ363254 | Microcystis aeruginosa | 1330 | 702 (100.00%) | 0 | 702 | 742.0 |
| 6 | D1 | AF139301 | Microcystis aeruginosa | 1421 | 702 (100.00%) | 0 | 702 | 742.0 |
| 7 | D1 | AY439281 | Microcystis sp. 205 | 1449 | 702 (100.00%) | 0 | 702 | 742.0 |
| 8 | D1 | AF139306 | Microcystis sp. UWOCC BauldB | 1421 | 702 (100.00%) | 0 | 702 | 742.0 |
| 9 | D1 | FJ461749 | Microcystis aeruginosa NIES-298 | 1481 | 702 (100.00%) | 0 | 702 | 742.0 |
| 10 | D1 | AB012338 | Microcystis ichthyoblabe | 1457 | 702 (100.00%) | 0 | 702 | 742.0 |
| 11 | D1 | AF139325 | Microcystis aeruginosa | 1421 | 702 (100.00%) | 0 | 702 | 742.0 |
| 12 | D1 | AF139321 | Microcystis aeruginosa | 1421 | 702 (100.00%) | 0 | 702 | 742.0 |
| 13 | D1 | FJ461750 | Microcystis aeruginosa NIES-101 | 1468 | 702 (100.00%) | 0 | 702 | 742.0 |
| 14 | D1 | AJ133172 | Microcystis sp. 199 | 1444 | 702 (100.00%) | 0 | 702 | 742.0 |
| 15 | D1 | AF139322 | Microcystis aeruginosa | 1421 | 702 (100.00%) | 0 | 702 | 742.0 |
| 16 | D1 | AF139324 | Microcystis aeruginosa | 1421 | 702 | 0 | 702 | 742.0 |

| **Signature:** | **Report Creator** | smulders |
|---|---|---|


Copyright (C) SmartGene 2001-2012

# Sequence-based Strain Identification Report

| **Sample name** | AMS 5-10-11 | **Bact code** | 101263 | **Project code** | 52436 |
|---|---|---|---|---|---|
| **Sample creation** | 18.07.2012 | **Sample update** | 18.07.2012 | | |

| Rank | Db | AC - Accession | OS - Organism | Seq. Length | Identities | Mismatches | Match Length | Score |
|---|---|---|---|---|---|---|---|---|
| | | | | | (100.00%) | | | |
| 17 | D1 | AF139326 | Microcystis aeruginosa | 1421 | 702 (100.00%) | 0 | 702 | 742.0 |
| 18 | D1 | FJ595690 | Microcystis sp. CHAB1442 | 1375 | 702 (100.00%) | 0 | 702 | 742.0 |
| 19 | D1 | DQ648030 | Microcystis aeruginosa UTEX 'LB 2388' | 1444 | 702 (100.00%) | 0 | 702 | 742.0 |
| 20 | D1 | GQ496076 | Microcystis smithii CHAB2183 | 1442 | 701 (99.86%) | 1 | 702 | 739.0 |
| 21 | D1 | AF139302 | Microcystis aeruginosa | 1421 | 701 (99.86%) | 1 | 702 | 739.0 |
| 22 | D1 | AF139317 | Microcystis aeruginosa | 1421 | 701 (99.86%) | 1 | 702 | 739.0 |
| 23 | D1 | AF139298 | Microcystis aeruginosa | 1421 | 701 (99.86%) | 1 | 702 | 739.0 |
| 24 | D1 | AF139307 | Microcystis sp. UWOCC CBS | 1421 | 701 (99.86%) | 1 | 702 | 739.0 |
| 25 | D1 | AF139295 | Microcystis aeruginosa | 1421 | 701 (99.86%) | 1 | 702 | 739.0 |
| 26 | D1 | FJ595696 | Microcystis sp. CHAB720 | 1375 | 701 (99.86%) | 1 | 702 | 739.0 |
| 27 | D1 | AF139297 | Microcystis aeruginosa | 1421 | 701 (99.86%) | 1 | 702 | 739.0 |
| 28 | D1 | AJ635433 | Microcystis ichthyoblabe 0BB39S02 | 1476 | 701 (99.86%) | 1 | 702 | 739.0 |
| 29 | D1 | EF121241 | Microcystis aeruginosa | 1477 | 701 (99.86%) | 1 | 702 | 739.0 |
| 30 | D1 | GQ496080 | Microcystis smithii CHAB2177 | 1443 | 701 (99.86%) | 1 | 702 | 739.0 |
| 31 | D1 | AM778951 | Microcystis aeruginosa PCC 7806 | 1492 | 701 (99.86%) | 1 | 702 | 739.0 |
| 32 | D1 | FJ595687 | Microcystis sp. CHAB1446 | 1375 | 701 (99.86%) | 1 | 702 | 739.0 |
| 33 | D1 | AF139312 | Microcystis sp. UWOCC BauldE | 1421 | 701 (99.86%) | 1 | 702 | 739.0 |
| 34 | D1 | FJ595697 | Microcystis sp. CHAB726 | 1375 | 701 (99.86%) | 1 | 702 | 739.0 |
| 35 | D1 | FJ595685 | Microcystis sp. CHAB729 | 1375 | 701 (99.86%) | 1 | 702 | 739.0 |
| 36 | D1 | AF139299 | Microcystis aeruginosa | 1421 | 701 (99.86%) | 1 | 702 | 739.0 |
| 37 | D1 | AM259270 | Microcystis sp. 1tu31s06 | 1464 | 701 (99.86%) | 1 | 702 | 739.0 |
| 38 | D1 | AJ133171 | Microcystis aeruginosa PCC 7941 | 1444 | 701 (99.86%) | 1 | 702 | 739.0 |

**Signature:** **Report Creator** smulders


Copyright (C) SmartGene 2001-2012

# Sequence-based Strain Identification Report

| **Sample name** | AMS 5-10-11 | **Bact code** | 101263 | **Project code** | 52436 |
|---|---|---|---|---|---|
| **Sample creation** | 18.07.2012 | **Sample update** | 18.07.2012 | | |

| Rank | Db | AC - Accession | OS - Organism | Seq. Length | Identities | Mismatches | Match Length | Score |
|---|---|---|---|---|---|---|---|---|
| 39 | D1 | FJ595686 | Microcystis sp. CHAB1444 | 1375 | 701 (99.86%) | 1 | 702 | 739.0 |
| 40 | D1 | FJ595698 | Microcystis sp. CHAB727 | 1375 | 701 (99.86%) | 1 | 702 | 739.0 |
| 41 | D1 | AF139319 | Microcystis aeruginosa | 1421 | 701 (99.86%) | 1 | 702 | 739.0 |
| 42 | D1 | AF139296 | Microcystis aeruginosa | 1421 | 701 (99.86%) | 1 | 702 | 739.0 |
| 43 | D1 | AF139318 | Microcystis aeruginosa | 1421 | 701 (99.86%) | 1 | 702 | 739.0 |
| 44 | D1 | AF139303 | Microcystis aeruginosa | 1421 | 701 (99.86%) | 1 | 702 | 739.0 |
| 45 | D1 | AF139327 | Microcystis flos-aquae | 1421 | 700 (99.72%) | 2 | 702 | 735.0 |
| 46 | D1 | AY439282 | Microcystis sp. GL260735 | 1449 | 700 (99.72%) | 2 | 702 | 735.0 |
| 47 | D1 | AF139310 | Microcystis sp. UWOCC MSU28-1 | 1421 | 700 (99.72%) | 2 | 702 | 735.0 |
| 48 | D1 | FJ595688 | Microcystis sp. CHAB1445 | 1375 | 700 (99.72%) | 2 | 702 | 735.0 |
| 49 | D1 | AB012333 | Microcystis aeruginosa | 1457 | 700 (99.72%) | 2 | 702 | 735.0 |
| 50 | D1 | AF139300 | Microcystis aeruginosa | 1421 | 700 (99.72%) | 2 | 702 | 735.0 |
| 51 | D1 | EU541973 | Microcystis novacekii MCYS-CH01 | 1404 | 700 (99.72%) | 2 | 702 | 735.0 |
| 52 | D1 | AB012330 | Microcystis sp. | 1457 | 700 (99.72%) | 2 | 702 | 735.0 |
| 53 | D1 | AF139313 | Microcystis aeruginosa | 1421 | 700 (99.72%) | 2 | 702 | 735.0 |
| 54 | D1 | AF139314 | Microcystis aeruginosa | 1421 | 700 (99.72%) | 2 | 702 | 735.0 |
| 55 | D1 | DQ648028 | Microcystis wesenbergii NIES-107 | 1444 | 700 (99.72%) | 2 | 702 | 735.0 |
| 56 | D1 | AB305067 | Microcystis aeruginosa | 1351 | 700 (99.72%) | 2 | 702 | 735.0 |
| 57 | D1 | AF139309 | Microcystis sp. UWOCC K | 1421 | 700 (99.72%) | 2 | 702 | 735.0 |
| 58 | D1 | DQ648026 | Microcystis aeruginosa NIES-90 | 1444 | 700 (99.72%) | 2 | 702 | 735.0 |
| 59 | D1 | AF139311 | Microcystis aeruginosa UWOCC Q | 1421 | 700 (99.72%) | 2 | 702 | 735.0 |
| 60 | D1 | AF139294 | Microcystis aeruginosa | 1421 | 700 (99.72%) | 2 | 702 | 735.0 |
| 61 | D1 | AF139293 | Microcystis aeruginosa | 1421 | 700 (99.72%) | 2 | 702 | 735.0 |

**Signature:** **Report Creator** smulders


Copyright (C) SmartGene 2001-2012

# Sequence-based Strain Identification Report

| **Sample name** | AMS 5-10-11 | **Bact code** | 101263 | **Project code** | 52436 |
|---|---|---|---|---|---|
| **Sample creation** | 18.07.2012 | **Sample update** | 18.07.2012 | | |

| Rank | Db | AC - Accession | OS - Organism | Seq. Length | Identities | Mismatches | Match Length | Score |
|---|---|---|---|---|---|---|---|---|
| 62 | D1 | AJ133170 | Microcystis sp. 130 | 1444 | 700 (99.72%) | 2 | 702 | 735.0 |
| 63 | D1 | FJ595689 | Microcystis sp. CHAB1449 | 1375 | 700 (99.72%) | 2 | 702 | 735.0 |
| 64 | D1 | U40338 | Microcystis aeruginosa | 1475 | 700 (99.72%) | 2 | 702 | 734.0 |
| 65 | D1 | AJ635432 | Microcystis aeruginosa 0BF29S03 | 1470 | 699 (99.57%) | 3 | 702 | 732.0 |
| 66 | D1 | AB012335 | Microcystis wesenbergii | 1457 | 699 (99.57%) | 3 | 702 | 732.0 |
| 67 | D1 | AB271211 | Microcystis aeruginosa | 1437 | 699 (99.57%) | 3 | 702 | 732.0 |
| 68 | D1 | AB012337 | Microcystis novacekii | 1457 | 699 (99.57%) | 3 | 702 | 732.0 |
| 69 | D1 | AB012329 | Microcystis sp. | 1457 | 699 (99.57%) | 3 | 702 | 732.0 |
| 70 | D1 | AB012327 | Microcystis sp. | 1457 | 699 (99.57%) | 3 | 702 | 732.0 |
| 71 | D1 | AM778928 | Microcystis aeruginosa PCC 7806 | 896 | 699 (99.57%) | 3 | 702 | 732.0 |
| 72 | D1 | FM177496 | Microcystis aeruginosa 2LT25S03 | 1461 | 699 (99.57%) | 3 | 702 | 732.0 |
| 73 | D1 | GQ496077 | Microcystis smithii CHAB139 | 1441 | 699 (99.57%) | 3 | 702 | 732.0 |
| 74 | D1 | AF139316 | Microcystis aeruginosa | 1421 | 699 (99.57%) | 3 | 702 | 732.0 |
| 75 | D1 | AB012326 | Microcystis sp. | 1457 | 699 (99.57%) | 3 | 702 | 732.0 |
| 76 | D1 | FJ595684 | Microcystis sp. CHAB1443 | 1375 | 699 (99.57%) | 3 | 702 | 732.0 |
| 77 | D1 | FM177498 | Microcystis ichthyoblabe 2LT25S02 | 1462 | 699 (99.57%) | 3 | 702 | 732.0 |
| 78 | D1 | AJ635431 | Microcystis aeruginosa 0BF29S01 | 1474 | 699 (99.57%) | 3 | 702 | 732.0 |
| 79 | D1 | AP009552 | Microcystis aeruginosa NIES-843 | 1492 | 699 (99.57%) | 3 | 702 | 732.0 |
| 80 | D1 | AJ635430 | Microcystis aeruginosa 0BB35S02 | 1483 | 699 (99.57%) | 3 | 702 | 732.0 |
| 81 | D1 | EU815063 | Microcystis aeruginosa NPCD-1 | 1413 | 699 (99.57%) | 3 | 702 | 732.0 |
| 82 | D1 | AB012340 | Microcystis aeruginosa | 1457 | 699 (99.57%) | 3 | 702 | 732.0 |
| 83 | D1 | FM177497 | Microcystis aeruginosa 2LT27S08 | 1462 | 699 (99.57%) | 3 | 702 | 732.0 |
| 84 | D1 | AB035553 | Microcystis wesenbergii | 1457 | 699 (99.57%) | 3 | 702 | 732.0 |

**Signature:** **Report Creator** smulders


Copyright (C) SmartGene 2001-2012

# Sequence-based Strain Identification Report

| **Sample name** | AMS 5-10-11 | **Bact code** | 101263 | **Project code** | 52436 |
|---|---|---|---|---|---|
| **Sample creation** | 18.07.2012 | **Sample update** | 18.07.2012 | | |

| Rank | Db | AC - Accession | OS - Organism | Seq. Length | Identities | Mismatches | Match Length | Score |
|---|---|---|---|---|---|---|---|---|
| 85 | D1 | FM177499 | Microcystis ichthyoblabe 2LT25S04 | 1462 | 699 (99.57%) | 3 | 702 | 732.0 |
| 86 | D1 | FJ595691 | Microcystis sp. CHAB728 | 1375 | 699 (99.57%) | 3 | 702 | 732.0 |
| 87 | D1 | AB012336 | Microcystis novacekii | 1457 | 698 (99.43%) | 4 | 702 | 729.0 |
| 88 | D1 | AJ133174 | Microcystis wesenbergii NIES-104 | 1446 | 698 (99.43%) | 4 | 702 | 729.0 |
| 89 | D1 | AF139315 | Microcystis aeruginosa | 1421 | 698 (99.43%) | 4 | 702 | 729.0 |
| 90 | D1 | AB035551 | Microcystis novacekii | 1457 | 698 (99.43%) | 4 | 702 | 729.0 |
| 91 | D1 | GQ496078 | Microcystis smithii CHAB110 | 1405 | 698 (99.43%) | 4 | 702 | 729.0 |
| 92 | D1 | AF139329 | Microcystis flos-aquae | 1421 | 698 (99.43%) | 4 | 702 | 729.0 |
| 93 | D1 | AF139328 | Microcystis flos-aquae | 1421 | 698 (99.43%) | 4 | 702 | 729.0 |
| 94 | D1 | AB035550 | Microcystis ichthyoblabe | 1457 | 698 (99.43%) | 4 | 702 | 729.0 |
| 95 | D1 | AJ133175 | Microcystis sp. 269 | 1443 | 698 (99.43%) | 4 | 702 | 727.0 |
| 96 | D1 | U03402 | Microcystis aeruginosa | 1411 | 698 (99.43%) | 4 | 702 | 727.0 |
| 97 | D1 | AJ133173 | Microcystis sp. GL280641 | 1443 | 698 (99.43%) | 4 | 702 | 727.0 |
| 98 | D1 | DQ648029 | Microcystis viridis NIES-1058 | 1444 | 697 (99.29%) | 5 | 702 | 725.0 |
| 99 | D1 | AJ635429 | Microcystis aeruginosa 1BB38S07 | 1473 | 697 (99.29%) | 5 | 702 | 725.0 |
| 100 | D1 | AB012328 | Microcystis viridis | 1457 | 697 (99.29%) | 5 | 702 | 725.0 |

**Signature:** **Report Creator** smulders


Copyright (C) SmartGene 2001-2012

BASECLEAR

FOR 100% DNA RESULTS

# Sequence-based Strain Identification Report

| **Sample name** | AMS 5-10-11 | **Bact code** | 101263 | **Project code** | 52436 |
|---|---|---|---|---|---|
| **Sample creation** | 18.07.2012 | **Sample update** | 18.07.2012 | | |

# Multi-alignment Excerpts

```
                                   1                                                                                        90
                                   ....:....|....:....|....:....|....:....|....:....|....:....|....:....|....:....|....:....|
AMS 5-10-11                      | ----------------------------------------TCTTCGGATTCTAGTGGCGGACGGGTGAGTAACGCGTAAGAATCTAACTT
Microcystis aeruginosa           | ACGCTGGCGGCGTGCCTAACACATGCAAGTCGAACGGGAA..................................................
Microcystis ichthyoblabe         | ACGCTGGCGGCGTGCCTAACACATGCAAGTCGAACGGGAA..................................................
Microcystis smithii CHAB2183     | ------CCCTTCCGCTTCTACCATGCA-GTCGAACGGGA-..................................................
                                                                           **************************************************


                                   91                                                                                      180
                                   ....:....|....:....|....:....|....:....|....:....|....:....|....:....|....:....|....:....|
AMS 5-10-11                      | CAGGACGGGGACAACAGTTGGAAACGACTGCTAATACCCGATGTGCCGCAAGGTGAAACCTAATTGGCCTGAAGAAGAGCTTGCGTCTGA
Microcystis aeruginosa           | ..........................................................................................
Microcystis ichthyoblabe         | ..........................................................................................
Microcystis smithii CHAB2183     | .......................................................................G..................
                                   *********************************************************************** ******************


                                   181                                                                                     270
                                   ....:....|....:....|....:....|....:....|....:....|....:....|....:....|....:....|....:....|
AMS 5-10-11                      | TTAGCTAGTTGGTGGGGTAAGAGCCTACCAAGGCGACGATCAGTAGCTGGTCTGAGAGGATGAGCAGCCACACTGGGACTGAGACACGGC
Microcystis aeruginosa           | ..........................................................................................
Microcystis ichthyoblabe         | ..........................................................................................
Microcystis smithii CHAB2183     | ..........................................................................................
                                   ******************************************************************************************


                                   271                                                                                     360
                                   ....:....|....:....|....:....|....:....|....:....|....:....|....:....|....:....|....:....|
AMS 5-10-11                      | CCAGACTCCTACGGGAGGCAGCAGTGGGGAATTTTCCGCAATGGGCGAAAGCCTGACGGAGCAACGCCGCGTGAGGGAGGAAGGTCTTTG
Microcystis aeruginosa           | ..........................................................................................
Microcystis ichthyoblabe         | ..........................................................................................
Microcystis smithii CHAB2183     | ..........................................................................................
                                   ******************************************************************************************


                                   361                                                                                     450
                                   ....:....|....:....|....:....|....:....|....:....|....:....|....:....|....:....|....:....|
AMS 5-10-11                      | GATTGTAAACCTCTTTTCTCAAGGAAGAAGTTCTGACGGTACTTGAGGAATCAGCCTCGGCTAACTCCGTGCCAGCAGCCGCGGTAATAC
Microcystis aeruginosa           | ..........................................................................................
Microcystis ichthyoblabe         | ..........................................................................................
Microcystis smithii CHAB2183     | ..........................................................................................
                                   ******************************************************************************************


                                   451                                                                                     540
                                   ....:....|....:....|....:....|....:....|....:....|....:....|....:....|....:....|....:....|
AMS 5-10-11                      | GGGGGAGGCAAGCGTTATCCGGAATTATTGGGCGTAAAGCGTCCGCAGGTGGTCAGCCAAGTCTGCCGTCAAATCAGGTTGCTTAACGAC
Microcystis aeruginosa           | ..........................................................................................
Microcystis ichthyoblabe         | ..........................................................................................
Microcystis smithii CHAB2183     | ..........................................................................................
                                   ******************************************************************************************


                                   541                                                                                     630
                                   ....:....|....:....|....:....|....:....|....:....|....:....|....:....|....:....|....:....|
AMS 5-10-11                      | CTAAAGGCGGTGGAAACTGGCAGACTAGAGAGCAGTAGGGGTAGCAGGAATTCCCAGTGTAGCGGTGAAATGCGTAGAGATTGGAAGAA
Microcystis aeruginosa           | ..........................................................................................
Microcystis ichthyoblabe         | ..........................................................................................
Microcystis smithii CHAB2183     | ..........................................................................................
                                   ******************************************************************************************
```

| **Signature:** | **Report Creator** | smulders |
|---|---|---|


Copyright (C) SmartGene 2001-2012

FOR 100% DNA RESULTS

# Sequence-based Strain Identification Report

| **Sample name** | AMS 5-10-11 | **Bact code** | 101263 | **Project code** | 52436 |
|---|---|---|---|---|---|
| **Sample creation** | 18.07.2012 | **Sample update** | 18.07.2012 | | |

```
                                 631                                                                                      720
                                 ....:....|....:....|....:....|....:....|....:....|....:....|....:....|....:....|....:....|
AMS 5-10-11                    | CATCGGTGGCGAAAGCGTGCTACTGGGCTGTATCTGACACTCAGGGACGAAAGCTAGGGGAGCGAAAGGGATTAGATACCCCTGTAGTCC
Microcystis aeruginosa         | ..........................................................................................
Microcystis ichthyoblabe       | ..........................................................................................
Microcystis smithii CHAB2183   | ..........................................................................................
                                 ******************************************************************************************
```

```
                                 721                                                                                      810
                                 ....:....|....:....|....:....|....:....|....:....|....:....|....:....|....:....|....:....|
AMS 5-10-11                    | TAGCCGTAAACGATGGATACTA--------------------------------------------------------------------
Microcystis aeruginosa         | ......................GGCGTGGCTTGTATCGACCCGAGCCGTGCCGAAGCTAACGCGTTAAGTATCCCGCCTGGGGAGTACGC
Microcystis ichthyoblabe       | ......................GGCGTGGCTTGTATCGACCCGAGCCGTGCCGAAGCTAACGCGTTAAGTATCCCGCCTGGGGAGTACGC
Microcystis smithii CHAB2183   | ......................GGCGTGGCTTGTATCGACCCGAGCCGTGCCGAAGCTAACGCGTTAAGTATCCCGCCTGGGGAGTACGC
                                 **********************
```

| **Signature:** | **Report Creator** | smulders |
|---|---|---|


Copyright (C) SmartGene 2001-2012

# Sequence-based Strain Identification Report

| | | | | | |
|---|---|---|---|---|---|
| **Sample name** | AMS 5-10-11 | **Bact code** | 101263 | **Project code** | 52436 |
| **Sample creation** | 18.07.2012 | **Sample update** | 18.07.2012 | | |

## Phylogenetic Tree

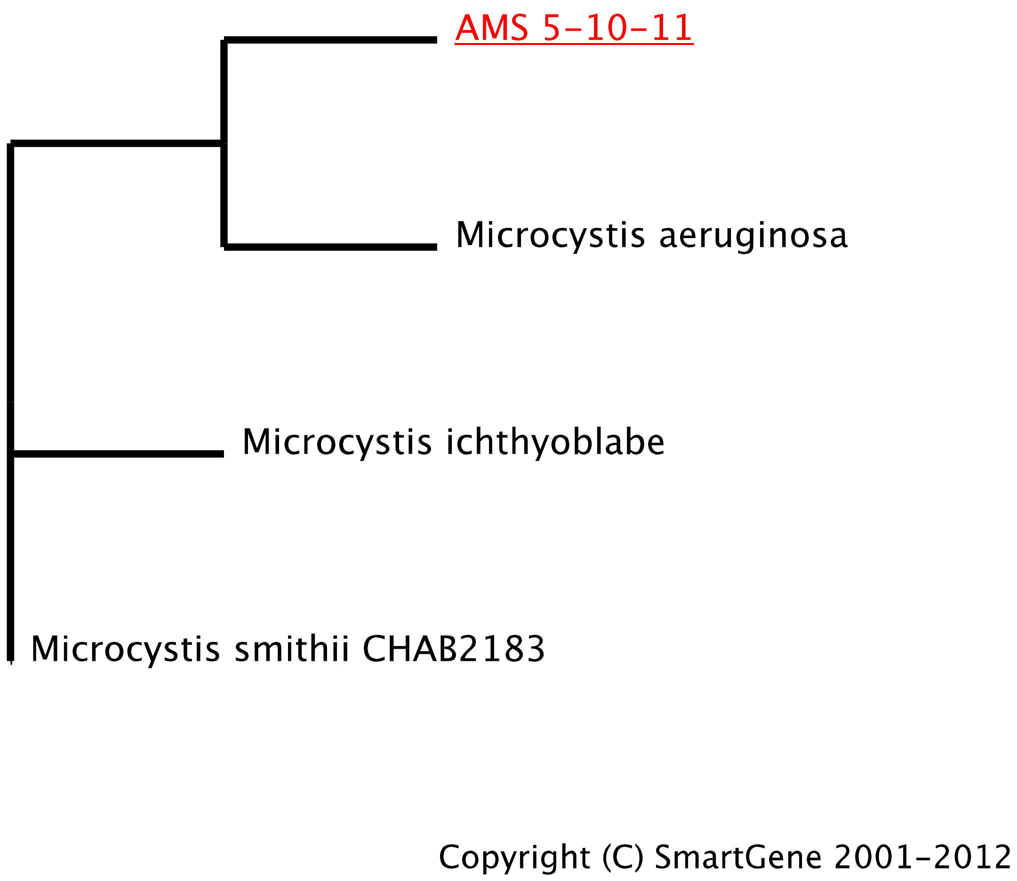



Copyright (C) SmartGene 2001-2012
