# Supplementary material for: Dog Poisonings Associated with a Microcystis aeruginosa Bloom in the Netherlands
Source: Toxins (Basel). 2013 Mar 14;5(3):556–67. doi: 10.3390/toxins5030556 (PMC3705278; doi:10.3390/toxins5030556)
Supplement: Supplementary File 2 — SI 2 Tables MC concentrations (PDF, 81 KB) [file toxins-05-00556-s003.pdf]

Supplementary Information

Table SI2.1. Microcystin concentration (μg L^{-1}) in eight water samples from Lake Amstelmeer. Values represent averages of analytical replicates and standard deviations, n = 3.

Sample	MC 7-dmRR		MC RR		MC YR		MC 7-dmLR		MC LR		MC LY		MC LW		MC LF	
	average	stdev	average	stdev	average	stdev	average	stdev	average	stdev	average	stdev	average	stdev	average	stdev
Location 1	2.2	0.00	51.1	0.49	5.1	0.05	282.4	2.72	2222.8	9.39	52.2	2.02	270.3	12.99	36.1	0.49
Location 2	2.1	0.03	62.6	0.78	4.5	0.03	224.2	1.98	2137.9	4.52	104.9	5.47	262.2	15.08	33.3	1.47
Location 3	0.2	0.00	3.4	0.06	1.1	0.03	0.6	0.02	10.6	0.13	0.2	0.01	0.6	0.03	0.2	0.01
Location 4	1.1	0.04	22.9	0.38	1.8	0.04	100.8	1.12	713.7	7.25	4.8	0.28	22.4	0.93	2.9	0.18
Location 5	1.6	0.04	34.5	0.06	13.1	0.19	10.4	0.05	98.0	0.23	3.7	0.44	9.2	0.57	3.8	0.16
Location 6	0.8	0.01	7.3	0.05	3.0	0.08	2.1	0.02	25.7	0.09	n.d.[1]	n.a.[2]	1.5	0.49	0.5	0.05
Location 7	2.1	0.04	45.3	0.86	24.6	0.05	20.9	0.18	226.2	0.24	3.0	0.13	7.4	0.25	2.2	0.06
Location 8	1.0	0.01	17.6	0.25	2.7	0.04	34.6	0.40	278.3	0.50	6.7	0.18	25.9	1.11	4.4	0.20

[1] not detected, [2] not applicable.

Table SI2.2. Microcystin concentration ($\mu g\ g^{-1}$ dry weight) in scum samples and dog vomit. Values represent averages of analytical replicates and standard deviations, $n = 3$.

Sample	MC 7-dmRR		MC RR		MC YR		MC 7-dmLR		MC LR		MC LY		MC LW		MC LF	
	average	stdev	average	stdev	average	stdev	average	stdev	average	stdev	average	stdev	average	stdev	average	stdev
Shore scum A	4.4	0.10	89.7	1.02	14.7	0.29	82.3	0.93	2124.9	6.42	81.6	2.93	242.6	5.21	80.3	2.37
Shore scum B	3.5	0.09	76.4	0.55	11.6	0.24	63.0	0.66	850.0	4.18	72.4	1.70	162.5	21.24	59.7	0.11
Shore scum C	3.6	0.03	71.8	0.25	11.3	0.28	60.3	0.23	631.0	7.04	67.4	3.90	199.9	12.12	53.9	0.90
Water scum I	28.2	0.37	571.4	9.98	268.2	5.37	572.2	9.36	3513.1	27.36	78.9	1.69	175.7	9.20	57.3	2.70
Water scum II	3.7	0.05	64.6	2.05	11.2	0.12	509.9	4.60	3864.1	8.38	114.8	3.55	270.4	25.99	92.7	3.67
Water scum III	3.6	0.11	61.6	0.60	9.2	0.09	56.8	0.80	1469.8	9.38	72.8	4.69	203.1	1.81	53.3	2.04
Vomit subsample x	0.5	0.01	6.0	0.12	0.5	0.01	2.4	0.02	109.7	1.23	0.8 [1]	n.a.[2]	4.2	0.05	1.2	0.16
Vomit subsample y	0.3	0.00	3.7	0.02	0.3	0.00	1.5	0.01	66.6	0.10	0.6	0.06	3.1	0.23	0.7	0.03
Vomit subsample z	0.2	0.00	3.0	0.02	0.3	0.01	1.4	0.02	70.2	0.41	0.6	0.06	2.8	0.44	0.8	0.02

[1] not detected in one replicate, value is average of two other replicates, [2] not applicable.

© 2013 by the authors; licensee MDPI, Basel, Switzerland. This article is an open access article distributed under the terms and conditions of the Creative Commons Attribution license (http://creativecommons.org/licenses/by/3.0/).
